# Mice that gorged during dietary restriction increased foraging related behaviors and differed in their macronutrient preference when released from restriction

**DOI:** 10.7717/peerj.1091

**Published:** 2015-07-02

**Authors:** Catherine Hambly, John R. Speakman

**Affiliations:** 1 Institute of Biological and Environmental Sciences, University of Aberdeen, Aberdeen, UK; 2 Institute of Genetics and Developmental Biology, Chinese Academy of Sciences, Beijing, China

**Keywords:** Gorging, Activity, Food restriction, Diet choice, Macronutrient

## Abstract

Caloric restriction (CR) can trigger gorging behavior. We examined macronutrient choice and behavior in mice that gorged during restriction compared to restricted non-gorgers and controls. Fifty MF1 male mice were restricted to 75% of *ad-libitum* food intake (FI), while ten controls were fed *ad-lib*. Body mass (BM) and FI were measured two and 24-h after food inclusion over 14-days. ‘Gorging’ mice were defined as those which ate over 25% of their daily FI in 2-h. The top 11 gorgers and the lowest 9 gorgers, along with 10 controls, had their behavior analysed during restriction, and were then provided with an unrestricted food choice, consisting of three diets that were high in fat, protein or carbohydrate. During restriction gorgers ate on average 51% of their daily FI in the 2-h following food introduction while the non-gorgers ate only 16%. Gorgers lost significantly more BM than non-gorgers possibly due to an increased physical activity linked to anticipation of daily food provision. Controls and non-gorgers spent most of their time sleeping. After restriction, both gorgers and non-gorgers were hyperphagic until their lost weight was regained. All 3 groups favoured high fat food. Gorgers and non-gorgers had a significantly greater high carbohydrate diet intake than controls, and gorgers also had a significantly greater high protein diet intake than non-gorgers and controls. On unrestricted food, they did not continue to gorge, although they still had a significantly greater 2-h FI than the other groups. Elevated protein intake may play an important role in the recovery of the lost lean tissue of gorgers after restriction.

## Introduction

Gorging (or bingeing) is characterised by the over consumption of food in a short period of time, and may be initially triggered by caloric restriction as is seen in human eating disorders (Corwin & Buda-Levin, 2004). Gorging can also develop as a consequence of a stressful event (Boggiano et al., 2007; Chandler-Laney et al., 2007; Gluck, 2006; Razzoli, Sanghez & Bartolomucci, 2015) but may only be displayed in the presence of palatable foods (Hagan et al., 2002). Hagan et al. (2002) found that combinations of caloric restriction (CR) or no restriction, and stress (foot shock) or no stress, did not promote gorging behavior on standard laboratory chow in rats, however when palatable food was also present, only the group that received both shock and restriction began gorging. Rats that had experienced caloric restriction and stress needed only a small amount of highly palatable food to trigger gorge eating of their normal chow diet (Hagan et al., 2003). This is similar to the human situation of binge eating where the sight or smell of a highly palatable food can cause binging episodes in satiated individuals (Rogers & Hill, 1989; Van der Ster Wallin, Norring & Holmgren, 1994; Walters, Hill & Waller, 2001). In rats, it has been found that orexin signalling may play a key role in this behaviour (Alcaraz-Iborra et al., 2014) and that it is more prevalent in adolescents compared to adults (Bekker et al., 2014) and females compared to males (Klump et al., 2013).

Macronutrients may also influence gorging. It has been shown that high protein meals produce greater levels of satiety when compared to food with a high fat or carbohydrate content (Astrup, 2005; Hill & Blundell, 1986; Paddon-Jones et al., 2008). Humans with Binge Eating Disorder or Bulimia Nervosa consume less protein during binging episodes, than times when they were not binging (Van der Ster Wallin, Norring & Holmgren, 1994) and they often start binges with desert and snack foods which are low in protein (Hadigan, Kissileff & Walsh, 1989). If bingers have a protein pre-load before they binge, the amount of food consumed is significantly reduced (Latner, 2003; Latner & Wilson, 2004). This is not true for preloads of carbohydrate or fat (Rolls et al., 1997). The food type chosen by gorgers during binging episodes could play a role in the amount of food craved. In both rats and humans, protein is currently believed to provide the greatest appetite suppression out of the three macronutrients (Bensaïd et al., 2002).

When mice are placed on caloric restriction they vary in the extent to which they gorge. In a previous study we showed that mice that gorged during restriction were less able to compensate for the reduced energy intake than non-gorgers (Hambly et al., 2007) and consequently lost more weight. The gorgers had high activity levels unlike the non-gorgers that showed a decline in activity. Although we have previously ascertained that gorging mice have a greater activity than non-gorgers, we were unable to quantify which behaviors were more greatly expressed (Hambly et al., 2007). In this previous study, we also did not explore the macronutrient preferences of the gorging and non-gorging mice when released from the dietary restriction. The aim of this study was therefore two-fold. First we aimed to determine the precise differences in behavior of the gorging and non-gorging mice during restriction and second to examine if there were any differences in macronutrient choice between *ad lib* fed controls, and mice that had developed gorging or non-gorging tendencies during restriction.

## Materials and Methods

All work was conducted under UK Home Office Licence 60/2881 and conformed to the UK Animals Scientific Procedures Act 1986.

### Baseline

Sixty male MF1 mice (Harlan UK Limited, England), aged 14 weeks, were used in this study. After a period of acclimation they were separated into individual cages (M3 cage 48 × 15 × 13 cm; NKP Cages, Kent, UK), each with constant access to water. The mice were maintained at a temperature of 21 °C ± 0.5 °C under a photoperiod of 12 h light/dark. They had sawdust bedding and a cardboard tunnel for enrichment.

For the first 14 days of the study all of the 60 mice were provided with a weighed amount of approximately 10 g of standard chow, (CRM; Special Diets Services, BP Nutrition, UK), which is in excess of their daily dietary requirements. The food was placed directly onto the cage floor at the same time each day (middle of their light period), to avoid difficulties obtaining it from the hopper. Each mouse and its food were weighed every day at the time of food introduction and then 2 h later. Any remaining food from the previous day was removed from the bedding and weighed before the next day’s food was given. Previous studies with this strain of mouse on this diet indicated that loss of minor food items in the sawdust amounted to less than 2% of daily intake (Johnson, Thomson & Speakman, 2001).

### Caloric restriction

From the 60 mice, 10 controls were selected which were matched for body mass with the remaining mice and these individuals continued to feed *ad lib* using the same regime as previously described. They were always fed at the same time as the restricted mice during the light phase. The remaining 50 mice were fed a restricted diet, calculated at 75% of each individuals average daily intake from the previous two weeks. As each individual was restricted according to its own baseline food intake, the level of treatment for all mice was therefore identical even although there will be variation between individuals in absolute terms. This diet restriction was continued for another 14 days, still with all 60 mice being weighed when the food was added and also 2 h later along with the remaining food. Near the end of the restriction period, we selected the top and bottom gorgers from the 50 restricted mice (*n* = 11 for gorgers and *n* = 9 for non-gorgers), and the other mice were removed from the study. Gorgers were determined as those that ate greater than 25% of their average daily intake in the first two hours after inclusion.

To assess the detailed behavior of the mice during the restriction period, we recorded footage of a subset of the mice. Three control mice, 3 mice showing gorging behavior and 3 mice showing non-gorging behavior were placed with their bedding in new cages which were identical in size to their previous cages (also M3 cage 48 × 15 × 13 cm; NKP Cages, Kent, UK) but were clear perspex with a dark screen behind them. Cardboard tubes were replaced with clear plastic igloos. After a 2 h period of acclimation to the new cage, a Hi-8 video camera (Sony CCD-VX1E/PAL) was used to record the mice for 3 h (one hour before food inclusion and two hours after food inclusion). While filming, the mice underwent the same regime as usual and were therefore weighed at the time of food inclusion, and two hours after as normal. Control mice also underwent their usual regime and had their food replaced at the same time as the restricted mice received their rations and were also weighed. The three-hour videos were converted to digital format and then analysed using HomeCageScan™ 2.0 (Clever Sys Inc., Virginia, USA). This software enabled a detailed analysis of the behaviours that mice conducted in the cage and has been validated to be over 90% accurate with respect to human scoring. The behaviour determined in each frame was recorded at a rate for 30 frames per second.

### Diet choice recovery

For the final 14 days, the eleven gorgers, nine non-gorgers and ten controls were put into larger individual cages (1290D polypropylene cage, 42.5 × 26.6 × 15 cm; Techniplast) to allow for the provision of a diet choice. The food hoppers of the cages were divided into three separate compartments, where we placed 20 g of each of the three different foods. The three food choices were high in fat, protein and carbohydrate (high protein DO4080301, high fat D12492 and high carbohydrate D12450B; Research Diets, New Brunswick, USA). The feeding regime remained unaltered with the 30 mice weighed before 20 g of each food was added to the hopper and then the mouse and each diet was reweighed at 2 and 24 h after food inclusion.

To calculate the water and gross energy content of the four different food types used in this study, a sample of each diet was dried to constant mass and then analysed using bomb calorimetry (Adiabatic bomb calorimeter; Gallenkamp, Loughborough, UK) (Table 1).

**Table 1:** Breakdown of the four different diet types used in this study as provided by Special Diets Services and Research Diets. Digestive efficiencies were provided by J Kagya-Agyeman (2009, unpublished data). The protein source was casein, fat source was lard and carbohydrate source was a combination of corn starch, maltodextrin and sucrose.

	Standard CRM	Carbohydrate	Fat	Protein
Product code	801722	D12450B	D12492	DO4080301
% Fat	9	10	60	10
% Protein	22	20	20	60
% Carbohydrate	69	70	20	30
Digestive efficiency %	74.9	92.2	87.4	92.9
Gross energy (kJ/g dry)	17.35	17.80	23.10	19.94

### Statistics

Normality tests (Anderson-darling) were completed on all data, which were normalised if required using a Box–Cox transformation before being subjected to ANOVA (Tukey’s), paired *t*-tests or General Linear Modelling (GLM). Minitab V16 (Minitab Inc, USA) was used throughout. Means are shown ± standard errors. The mean of the last 5 days of each time period, baseline, restriction, and diet choice are presented unless otherwise stated.

## Results

### Baseline

Only data collected from the animals that were later selected as gorgers (*n* = 11), non-gorgers (*n* = 9) and controls (*n* = 10) was analysed. During the baseline period the mice that subsequently went on to become gorgers ate 0.5 g less than the other 2 groups (ANOVA *F*_2,27_ = 9.07, *P* = 0.001). This meant that their energy intake was also significantly lower (Table 2). During baseline however there were no significant differences between the three groups for dry food intake two hours after introduction of food (ANOVA *F*_2_, _27_ = 2.16, *P* = 0.14), and the mice ate an average of 2.9% of their total food intake within this time with a range of 0.4–7.8% indicating that they did not show any gorging tendencies prior to restriction. Body mass was not significantly different between the 3 groups and the rate of BM increase was on average 0.15 g/day which was not significantly different between the 3 groups even although there was a significant difference in daily food intake.

**Table 2:** The average measurements for the three different groups of mice during baseline, restriction and while on diet choice. Data are shown ± standard errors and are averaged for each individual for the final 5 days of each phase of the study unless otherwise stated.

	Gorger (*n* = 11)	Non-Gorger (*n* = 9)	Control (*n* = 10)
**Baseline**			
24 h dry food intake (g)	5.5 ± 0.12^a^	6.0 ± 0.10^b^	5.9 ± 0.06^b^
2 h dry food intake (g)	0.22 ± 0.03^a^	0.17 ± 0.03^a^	0.14 ± 0.03^a^
% of total food intake in 2 h	4.0 ± 0.50^a^	2.7 ± 0.42^a^	2.3 ± 0.54^a^
Energy intake (kJ/day)	95.0 ± 2.10^a^	104.3 ± 1.75^b^	103.5 ± 2.10^b^
Energy assimilated (kJ/day)	72.9 ± 1.96^a^	80.0 ± 1.96^b^	78.5 ± 2.09^ab^
Body mass (g)	38.2 ± 0.71^a^	38.7 ± 0.69^a^	36.4 ± 0.80^a^
**Restriction**			
24 h dry food intake (g)	4.1 ± 0.07^a^	4.5 ± 0.09^a^	6.7 ± 0.21^b^
2 h dry food intake (g)	2.11 ± 0.23^a^	0.70 ± 0.09^b^	0.11 ± 0.03^c^
% of total food intake in 2 h	51.6 ± 5.98^a^	15.8 ± 1.98^b^	1.5 ± 0.33^c^
Energy intake (kJ/day)	71.4 ± 1.26^a^	77.9 ± 1.51^a^	116.0 ± 3.71^b^
Energy assimilated (kJ/day)	53.5 ± 0.94^a^	58.32 ± 2.78^a^	86.1 ± 1.13^b^
Body mass (g)	34.9 ± 0.96^a^	37.5 ± 0.72^a^	38.0 ± 1.08^a^
**Diet choice (first 3 days)**			
24 h dry food intake (g)	5.8 ± 0.28^a^	5.0 ± 0.35^ab^	4.1 ± 0.20^b^
2 h dry food intake (g)	0.83 ± 0.11^a^	0.34 ± 0.06^b^	0.15 ± 0.03^b^
% of total food intake in 2 h	14.0 ± 1.62^a^	6.6 ± 0.93^b^	3.6 ± 0.63^b^
Energy intake (kJ/day)	121.2 ± 4.80^a^	102.6 ± 6.31^b^	89.9 ± 3.74^b^
Energy assimilated (kJ/day)	97.6 ± 2.48^a^	88.8 ± 78.04^ab^	78.0 ± 1.82^b^
Body mass (g)	39.5 ± 0.92^a^	39.6 ± 0.95^a^	38.5 ± 1.19^a^
Combined protein intake (kJ/day)	25.8 ± 0.60^a^	20.7 ± 1.26^b^	18.0 ± 0.76^b^
Combined fat intake (kJ/day)	50.3 ± 1.95^a^	41.5 ± 2.99^b^	46.5 ± 2.20^ab^
Combined carbohydrate intake (kJ/day)	45.1 ± 4.06^a^	40.36 ± 4.83^a^	25.4 ± 2.94^b^
**Diet choice (last 5 days)**			
24 h dry food intake (g)	3.8 ± 0.07^a^	3.7 ± 0.08^a^	3.8 ± 0.13^a^
2 h dry food intake (g)	0.18 ± 0.02^a^	0.09 ± 0.02^b^	0.09 ± 0.02^b^
% of total food intake in 2 h	4.6 ± 0.62^a^	2.5 ± 0.41^b^	2.4 ± 0.57^b^
Energy intake (kJ/day)	82.1 ± 1.60^a^	78.7 ± 2.17^a^	84.8 ± 2.85^a^
Energy assimilated (kJ/day)	72.8 ± 1.37^a^	69.7 ± 2.49^a^	74.7 ± 1.80^a^
Body mass (g)	41.6 ± 1.02^a^	41.8 ± 0.96^a^	41.2 ± 1.18^a^

**Notes.**

Values with common superscripts are not significantly different between the groups at the same time point.

### Restriction phase

**Figure 1:**
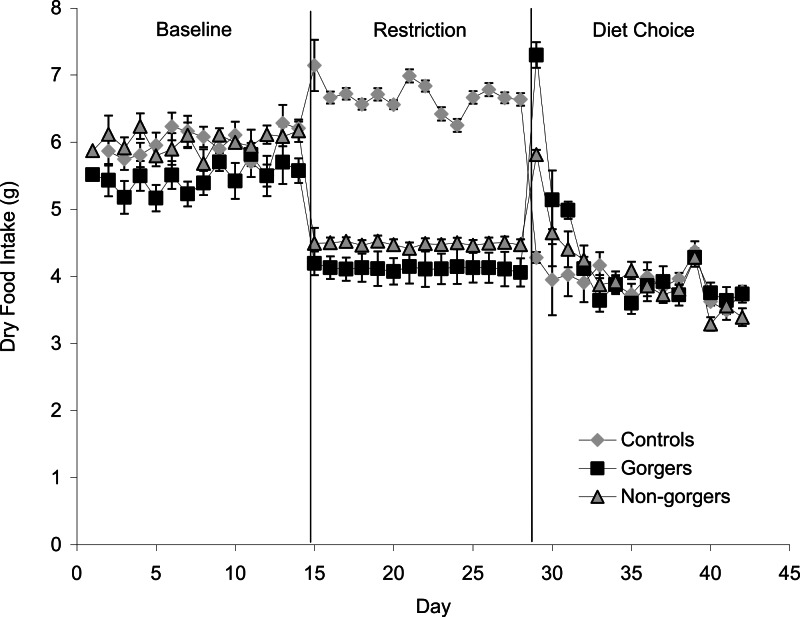
Mean daily dry food intake (g/day) during baseline, restriction and diet choice for the three groups (controls, gorgers and non-gorgers). The diet choice period is the combined intake for high fat, protein and carbohydrate diets. Standard error bars are shown.

**Figure 2:**
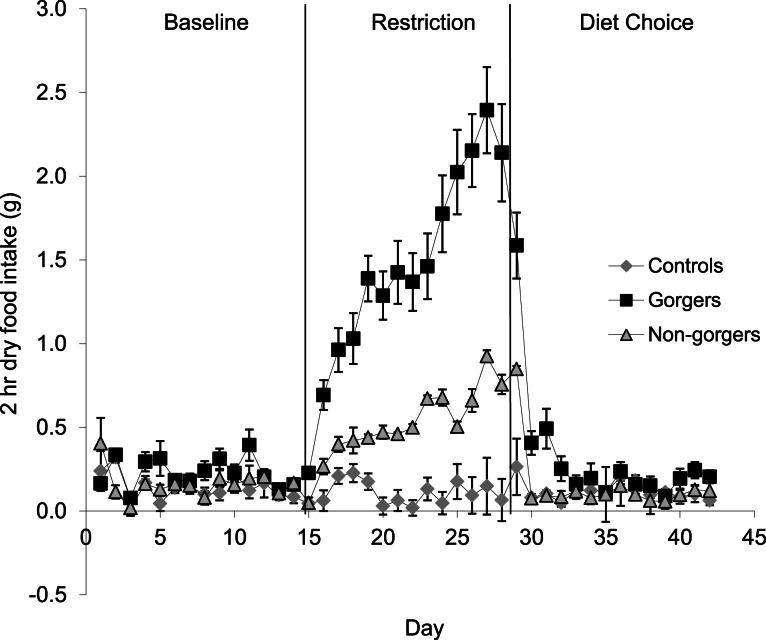
Mean dry food intake (g/day) during a two-hour period after food inclusion for baseline, restriction and diet choice. The diet choice period is the combined intake for high fat, protein and carbohydrate diets. Standard error bars are shown. Gorging mice ate an average of 51.6% ± 5.98% of their total food intake in 2 h during restriction, non-gorgers ate 15.8% ± 1.98%, while controls only ate 1.5 ± 0.33%. Gorging behaviour did not continue after restriction.

Each restricted mouse received exactly 25% less food than it consumed when provided with food *ad lib* which was, as expected, a significant reduction in food and energy intake (Fig. 1). There was a significant increase in the daily food intake of control mice over the same time period as the restriction by an average of 0.72 ± 0.19 g/day (10.8%) (Paired *t*-test *P* = 0.02). Consequently the realised restriction relative to controls averaged 35%. Mice that showed gorging behavior were apparent after only 3 days of restriction with significant increases in 2 h food intake above the control and non-gorging mice occurring in some individuals, even on day 1. The extent of gorging behavior increased throughout the 14 day measurement period so that the increase in 2 h food intake was highly significant when averaged over the last 5 days of the restriction (ANOVA *F*_2_, _27_ = 44.8, *P* < 0.001). Gorging mice ate an average of 51.6% ± 5.98% of their total food intake in 2 h, which was 3 times that eaten by non-gorgers in the same period and 21 times higher than the controls (Fig. 2). The ‘non-gorgers’ also increased their 2 h food intake during restriction but did not exceed the arbitrary limit to become defined as a gorger (25% of available food in 2 h) as they only consumed an average of 15.8% ± 1.98% of their total food intake in 2 h, which was significantly above the controls that ate 15 ± 0.33% of their 24 h food intake over the same 2 h period.

**Figure 3:**
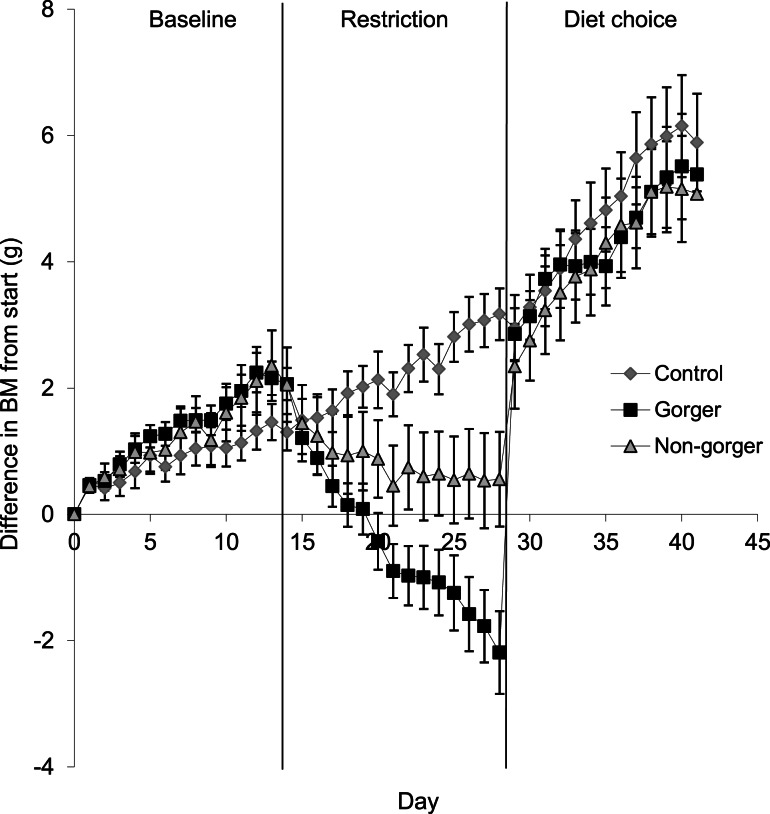
Change in body mass (g) from initial mass on day one of baseline over the course of baseline, restriction and diet choice. Standard error bars are shown.

To compare body mass of the three groups for each phase, the last five days were averaged for all animals in each group. Gorgers and non-gorgers both significantly decreased their body mass during restriction by an average of 3.3 g and 1.2 g respectively (Paired *t*-test *P* < 0.001). The decrease in body mass observed in the gorgers compared to their baseline value was significantly greater than the non-gorgers (9.6% compared to 3.4%). The controls significantly increased their body mass over the same time period by 1.6 g (4.4%) (Paired *t*-test *P* < 0.01) (Fig. 3). Over the last 5 days of restriction the gorgers were still losing weight at a rate of 0.22 ± 0.04 g/day while the non-gorgers had stabilised their body mass as the average rate of weight loss was only 0.04 ± 0.05 g/day showing that they had almost reached energy balance. In contrast the controls were gaining weight at 0.14 ± 0.04 g/day. These values were significantly different between the 3 groups (ANOVA *F*_2,27_ = 14.7, *P* < 0.001).

### Behavioral analysis

During the restriction phase, there was a significant difference in the behavior of the three groups. For the hour before food was introduced the controls spent a significantly greater amount of time sleeping than both gorgers and non-gorgers, while non-gorgers slept more than gorgers (ANOVA *F*_2,8_ = 50.8, *P* < 0.001). As expected, gorgers therefore showed greater amounts of general activity than the other 2 groups, (ANOVA *F*_2,8_ = 80.1, *P* < 0.001). This was accounted for by the fact that gorgers spent more time foraging (looking through the bedding and around the cage) than non-gorgers who in turn spent more time than the controls (ANOVA *F*_2,8_ = 3632.79, *P* < 0.001). Both restricted groups spent equal amounts of time climbing on the bars and rearing up on their hind legs which was more than the non-restricted controls (ANOVA climbing *F*_2,8_ = 5968.27, *P* < 0.001; rearing *F*_2,8_ = 14.23, *P* = 0.009). These behaviors both involve looking outside the cage, possibly to see when food would arrive. There was no difference in the amount of drinking or low level activity (general movement on the cage floor) between the groups (Table 3A).

**Table 3:** Mean behavior shown by restricted non-gorging, restricted gorging and *ad lib* control mice, (A) one hour before food inclusion and (B) 2 h after food inclusion (*n* = 3 per group). Data represents the percentage of time spent conducting a particular behavior as analysed using HomeCageScan™ 2.0. “Forage” includes searching through the bedding looking for food, “Remain low” is all other ambulatory activity that takes place in the bottom of the cage, “Reared” is standing up on its back legs, “Minor Behaviors” includes a combination of twitching, yawning, grooming and other short term intermittent behaviors which occur while the mouse is stationary.

1 h before %	Climb	Forage	Drink	Sleep	Reared	Remain low	Minor	Eat
**A**								
Control	0.7 ± 0.3	0.7 ± 0.2	0.04 ± 0.03	71.0 ± 4.3	3.6 ± 0.4	24.0 ± 3.5	0.0	0.0
Non Gorger	28.6 ± 0.8	7.0 ± 0.3	0.22 ± 0.21	28.2 ± 0.6	10.5 ± 0.2	21.0 ± 0.5	4.5 ± 3.0	0.0
Gorger	28.8 ± 1.4	32.6 ± 1.6	0.64 ± 0.6	0.0	11.7 ± 1.2	20.2 ± 0.5	6.0 ± 3.0	0.0

**2 h after %**	**Climb**	**Forage**	**Drink**	**Sleep**	**Reared**	**Remain low**	**Minor**	**Eat**
**B**								
Control	0.5 ± 0.3	0.0	0.04 ± 0.03	70.9 ± 4.6	0.8 ± 0.4	0.7 ± 0.3	26.6 ± 5.7	0.5 ± 0.01
Non Gorger	7.9 ± 1.9	0.4 ± 0.4	0.1 ± 0.07	36.5 ± 8.3	1.5 ± 0.4	4.2 ± 1.3	46.8 ± 13.2	2.7 ± 1.0
Gorger	13.0 ± 0.2	7.2 ± 0.3	1.4 ± 0.7	0.0	4.2 ± 0.4	10.7 ± 0.8	15.6 ± 5.9	47.9 ± 5.6

After food inclusion, controls spent a significantly greater amount of time sleeping than non-gorgers who in turn, slept more than gorgers (ANOVA *F*_2,8_ = 692.3, *P* < 0.001). Gorgers were more generally active spending a significantly greater amount of time foraging (ANOVA *F*_2,8_ = 71.85, *P* < 0.001), rearing (ANOVA *F*_2,8_ = 71.27, *P* = 0.006), eating (ANOVA *F*_2,8_ = 614.99, *P* < 0.001) and conducting low level activity (ANOVA *F*_2,8_ = 34.85, *P* < 0.001) than non-gorgers and controls who did not differ from each other in these activities (Table 3B). The differences between controls and non-gorgers was apparent when comparing climbing behavior as non-gorgers spent significantly more time climbing than the controls (ANOVA *F*_2,8_ = 47.08, *P* = 0.001). There was no difference in drinking activity between the three groups.

### Diet choice

**Figure 4:**
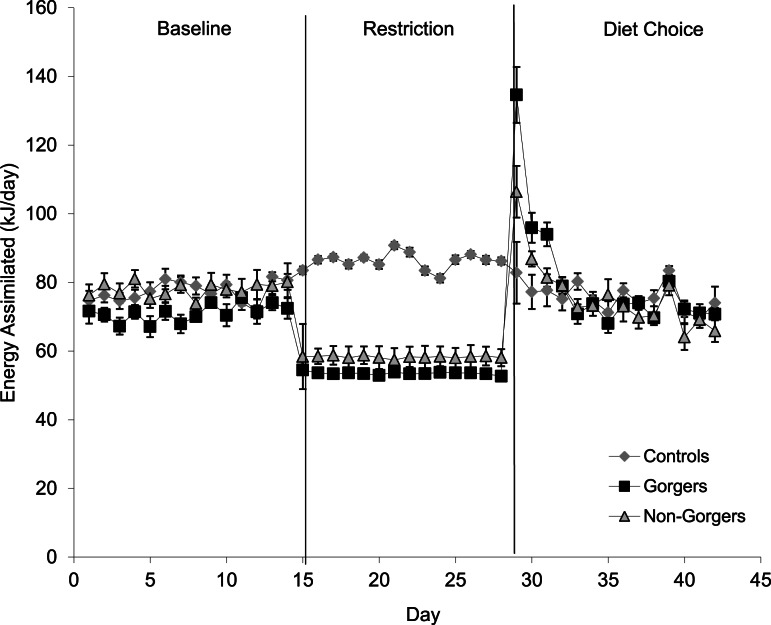
Mean energy assimilated (kJ/day) over a 24-h period for the three groups. The diet choice period is the combined assimilation for high fat, protein and carbohydrate diets. Standard error bars are shown.

After providing all three groups with a choice of diets high in fat, carbohydrate and protein, the controls daily food intake significantly decreased so that over the last 5 days of measurements it was 3.8 ± 0.13 g which was 2.1 g less than just prior to diet choice provision (Table 2). They ate some of all three diets and although the energy value for each diet was greater than the previously fed chow (Table 1), the energy intake calculated from the total dietary intake was reduced by 31.2 kJ/day (or 26.9% lower) than prior to providing the diet choice. This difference was significant (Paired *T*-test *T* = 5.50, *P* < 0.001). They managed, however, to maintain a similar rate of body mass increase even with the reduced energy intake. This is because there were increases in digestive efficiency on the 3 diets compared to the standard chow (Table 1 previously measured by J Kagya-Agyeman, 2009, unpublished data) which meant that the energy assimilated was not significantly different from the baseline period (Paired *t*-test *T* = 1.19, *P* = 0.26) (Fig. 4).

For the mice that had been subjected to dietary restriction, there was an initial marked hyperphagia over the first 3 days of free diet choice before food intake stabilised at a level that did not differ significantly from controls (ANOVA *F*_2,27_ = 0.96, *P* = 0.40). Mice that had been gorgers continued to have higher 2 h intake than either non-gorgers or controls when provided with the *ad lib* diet choice. On average they consumed over double that of the non-gorgers and controls in 2-h after feeding (ANOVA day 1–3 *F*_2,27_ = 23.28, *P* < 0.001). This pattern persisted throughout the 14 days of measurement after being given *ad lib* access to food (day 10–14: *F*_2,27_ = 5.73, *P* = 0.008). During day 1–3 of the diet choice, 24 h energy intake in the gorgers was significantly higher (25%) than their baseline energy intake (*P* < 0.001). Non-gorgers, however, matched their energy intake to that of the baseline period (Paired *T*-test *T* = 0.25, *P* = 0.81). Both the gorgers and non-gorgers fully recovered their lost body mass in the first few days (Fig. 3) so that they were not different to the controls (ANOVA *F*_2,27_ = 0.10, *P* = 0.91). BM was above the initial baseline measurements for the gorgers (Paired *T*-test *T* = 4.04, *P* = 0.002) although did not reach significance for the non-gorgers (Paired *t*-test *T* = 2.21, *P* = 0.058). The energy intake over the final 5 days of diet choice, like the controls, was significantly lower than the baseline measurement for these 2 groups (Paired *T*-test *P* < 0.001) although they still maintained a positive weight gain at the same rate as during the baseline period. As with the controls this was due to the increased digestive efficiency resulting in an energy assimilation which was not significantly different than baseline for gorgers (Paired *T*-test *T* = 0.01, *P* = 0.99). The non-gorgers had a slight but significant reduction in assimilated energy by 10 kJ/day (13%) relative to baseline (Paired *T*-test *T* = 3.37, *P* = 0.01).

**Figure 5:**
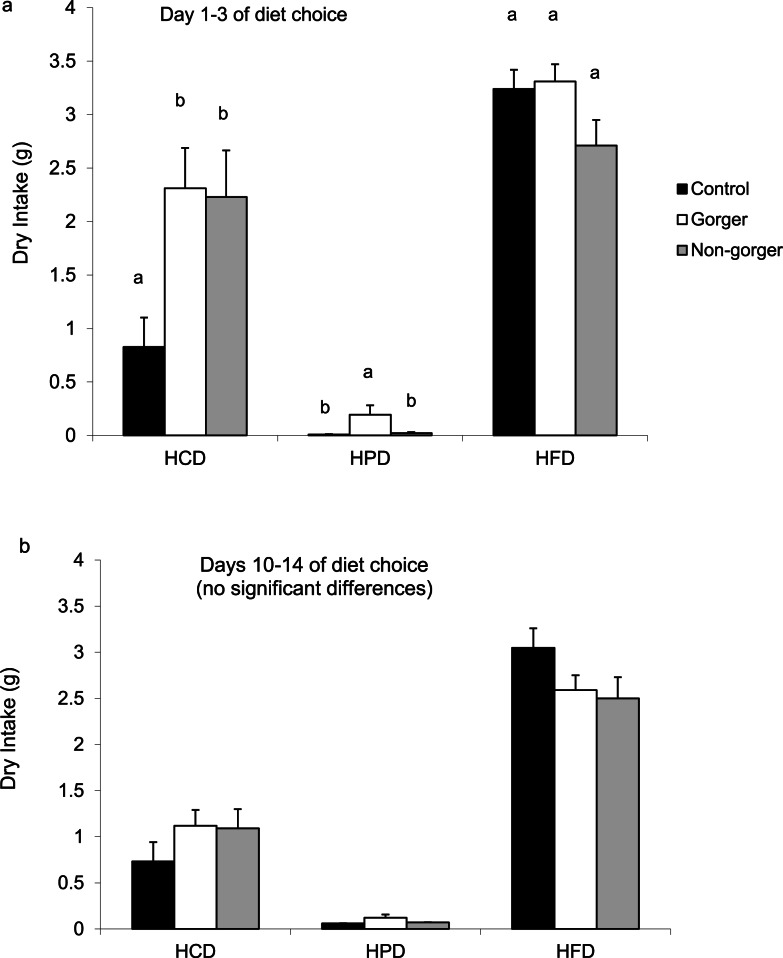
Mean daily intake of the three nutrients, high carbohydrate diet (HCD), high protein diet (HPD) and high fat diet (HFD) by the three groups of mice during days (A) 1–3 and (B) 10–14 of diet choice. Standard error bars are shown. Bars with common letters show no significant difference between the groups for each diet category.

The different amounts of each diet consumed (high protein, high fat or high carbohydrate) over either the first 1–3 days or the last 5 days of diet choice were compared. In all groups, the high fat diet was preferred above the high carbohydrate diet and least preferred was the high protein diet (Fig. 5). Both groups of animals that had been on food restriction had a significantly greater intake of high carbohydrate diet than controls over the first 3 days (ANOVA *F*_2,27_ = 5.19, *P* = 0.012; Fig. 5A). In addition, the gorgers also had a significantly greater intake of high protein diet than both non-gorgers and controls (ANOVA *F*_2,27_ = 3.70, *P* = 0.038). High fat diet consumption didn’t differ between the groups. The amount of energy consumed for each of the three macronutrients across all 3 diets was calculated (Table 2). The restricted mice did consume more carbohydrate when combining all 3 diets than the controls while only the gorging mice increased their intake of protein. By the end of the diet choice period, the intakes of each diet had normalised to the levels of the controls (Fig. 5B).

## Discussion

There was a huge variation in the extent that individuals in this study gorged, ranging between 4% and 79% of the daily ration consumed within 2 h of food provision in the restricted mice. This was despite them all receiving the same individual level of restriction. During restriction non-gorging mice were able to compensate more effectively for reduced caloric intake than gorging mice and therefore did not lose as much weight, which is similar to our previous study (Hambly et al., 2007). Reduced caloric intake can trigger both physiological compensatory responses (Guppy & Withers, 1999; Hambly et al., 2007) as well as changes in behavior (Hambly et al., 2005; Overton & Wilson, 2004). Greater weight loss has been found in mice bred for high activity compared to a low activity control line when both were placed on CR (Smyers et al., 2015). Similarly we previously observed that activity levels were much greater in gorging mice compared to non-gorging mice (Hambly et al., 2007). More detailed analysis in the current study allowed us to quantify the changes in behavior. Gorging mice specifically increased food anticipatory behaviors prior to feeding and spent more time eating after food became available than controls and non-gorgers as expected. It has been suggested that caloric restriction can also trigger periods of spontaneous activity (Overton & Wilson, 2004), which is particularly evident when rodents are calorically restricted and provided with a running wheel. This increased running wheel activity has been shown to stop on the first day of *ad lib* refeeding suggesting that the spontaneous activity ceases when the stimulus is removed (Hebebrand et al., 2003).

Both restricted groups showed clear food anticipatory behaviour in the hour prior to feeding however it was more pronounced in the gorging group. The gorgers spent all of their time in active behaviors while the non-gorgers spent 72% and controls spent 29% of their time being active. In particular, foraging, climbing and rearing behavior indicate that the mouse may be looking outside the cage to determine when its food will be provided. Controls only conducted this type of activity 5% of the time in comparison to 73% in gorgers and 46% in non-gorgers. The development of food anticipatory behavior during caloric restriction in rodents is well documented. Daily rhythms in behaviour and physiology are controlled by circadian clocks. As mice are nocturnal and have peak feeding activity around dusk and dawn, they had to reset their clocks in this study to accommodate the daylight feeding. The driver for circadian rhythms is the master pacemaker in the suprachiasmatic nuclei (SCN) of the hypothalamus (Mendoza, 2007) which coordinates other oscillators through neural, hormonal and behavioral signals (Dibner, Schibler & Albrecht, 2010). However, when changes in the time of food availability combine with caloric restriction, behavioural and physiological circadian rhythms shift usually without alternation in the phases of the SCN which is entrained on the light/dark cycle (Stokkan et al., 2000). The restricted mice in this study were able to predict meal time, and this process is thought to be under the control of a food-entrainable oscillator (FEO) (Feillet, Albrecht & Challet, 2006). Many brain regions and peripheral signals have been examined for a role in food anticipatory behavior, but thus far none have been identified as essential to the process (Mistlberger, 2011; Davidson, 2009). Leptin and the melanocortin pathway is more recently thought to exert control over locomotor behaviour (Ceccarini et al., 2015). The melanocortin system is found in the hypothalamic structures required for food entrainment. Food anticipatory activity has been assessed in wild-type (WT) and melanocortin-3 receptor-deficient (Mc3r-/-) mice (Sutton et al., 2008). WT mice showed increased wheel activity during the 2 h prior to feeding a restricted meal however, the food anticipatory response was reduced in mice lacking Mc3r suggesting an active role (Sutton et al., 2008). In addition, peripheral clocks could use hormonal pathways to entrain central FEOs. Potential hormones include leptin, insulin, ghrelin and corticosterone but again, none of these has a strong influence on its own suggesting a complex and elusive mechanism is involved (Patton & Mistlberger, 2013). The extent or speed of onset of food anticipatory behaviour has been found to depend on the extent of restriction (Gallardo et al., 2014). In mice, a restriction of 60% compared to lesser restrictions led to anticipatory behavior starting more quickly and reaching greater levels (Gallardo et al., 2014). In our study we saw a variation in the extent of this behaviour despite all animals receiving the same level of individual restriction so there clearly must be more triggering the variation in response.

The weight gain after restriction by both gorgers and non-gorgers was extremely rapid, due to the large increase in energy intake above that of controls over the first 3 days of macronutrient choice. It is also likely that the immediate weight gain was at least partially due to increased gut fill, but as the hyperphagia subsided it will have been replaced by gains in lean and fat tissue. Hyperphagia and weight regain after restriction is a well researched area in both humans and animal models involving leptin and the hypothalamic pathways NPY/AGRP and POMC/CART (Hambly et al., 2012). As gorgers had lost more weight under restriction, this may explain why their hyperphagia was more pronounced. The gorging mice lost 9.6% of their body mass while the non-gorgers only lost 3.4% and so had substantially more tissue to recover. Previous restrictions studies on this strain and sex of mouse suggest that weight loss under restriction consists of about 60% losses in lean tissue and 40% losses in fat (Hambly et al., 2012) and so the diets they selected may have differed to promote rapid lean tissue recovery. During the period of macronutrient choice, for the first 3 days of hyperphagia, the gorgers chose to eat more protein than controls and non-gorgers and both of the restricted groups consumed greater levels of carbohydrate than controls. Gorgers, for example, that lost larger amounts both lean and fat tissue consumed more protein than the other groups which may have enabled their lean tissue recovery to be maximised (e.g., Dulloo, Jacquet & Girardier, 1997). Similarly an obligate carnivore, the mink (*Neovison vison*), was found to be able to compensate for a period of nutritional imbalance by regulating their intake of macronutrients to match their requirements (Jensen et al., 2014). After day three of diet choice, the three groups had no significant difference between their energy intakes, which coincided with the time at which the restricted mice had recovered their lost weight.

Non-restricted controls showed reduced food intake when given the *ad lib* diet choice, however the total energy that they assimilated matched the previous diet they consumed due to differences in energy assimilation efficiency. We previously found that the MF1 strain of mouse adjusted their food intake when fed a high fat diet to compensate for the greater energy density (Hambly et al., 2005). These mice were able to exactly match the amount of energy they assimilated on a high fat compared to a low fat diet which allowed them to maintain a stable body mass. Similarly, the mice in the current study did not continue to gorge, and rapidly matched the amount of energy assimilation to what they consumed prior to restriction. Despite retaining a slightly higher 2 h food intake, the gorgers daily energy assimilation levels matched the 2 other groups so they did not become obese after release from caloric restriction. This strain of mouse is therefore adept at matching its nutritional and energetic requirements when provided with a choice of diets, as has been found in domestic cats (Hewson-Hughes et al., 2013).

In conclusion, the present results supported our previous data, which showed mice that developed gorging behavior were less able to compensate for caloric restriction than non-gorgers due to a difference in activity levels. This study however was able to quantify the extent of the different behaviours which were conducted by the mice and highlighted their increased food anticipatory behaviour prior to feeding. After restriction both gorgers and non-gorgers showed hyperphagic behavior, however it only lasted until the lost weight was regained. The groups did, however, show preferences for different food types over the first 3 days of the recovery *ad lib* period. Both restricted groups consumed greater amounts of carbohydrate than the controls but in addition, the gorging mice also consumed greater amounts of protein than the other 2 groups.

## Supplemental Information

10.7717/peerj.1091/supp-1 Supplemental Information 1 All Data Excel spread sheet containing dry food intakes, energy intakes, macronutrient choice and activity data.
